# Supplementary material for: Glycemic Index of Slowly Digestible Carbohydrate Alone and in Powdered Drink-Mix
Source: Nutrients. 2019 May 29;11(6):1228. doi: 10.3390/nu11061228 (PMC6627922; doi:10.3390/nu11061228)
Supplement: Supplementary file 1 [file nutrients-11-01228-s001.pdf]

Glycemic Index of Slowly Digestible Carbohydrate Alone and in Powdered Drink-Mix
Manuscript ID: nutrients-473016

Supplementary Table: Powdered Drink-mix Formulation

Ingredients	Control drink-mix (g)	SDC drink-mix (g)
Milk protein concentrate	4.13	4.26
**Maltodextrin**	**11.6**	**5.21**
Buttermilk powder	3.21	3.31
Sunflower oil powder	2.75	2.84
**Sugar (white, granulated)**	**12.64**	**5.53**
Soy Lecithin powder	0.46	0.47
Flavor (Chocolate extract)	0.68	0.71
Salt	0.09	0.09
Flavor (Vanillin)	0.09	0.09
Cocoa powder	8.27	8.52
Gum/Hydrocolloid system	1.83	1.89
Sweetener	0.09	0.09
**SUSTRA™ SDC**	**0.0**	**14. 31**

SDC: Slowly digestible carbohydrate
